# A Systematic Review of Clinical and Experimental Periodontitis Studies Demonstrating the Expression of PPAR-Gamma: A Meta-Analysis and Bioinformatics Approach

**DOI:** 10.3390/biomedicines13082028

**Published:** 2025-08-20

**Authors:** Marco Antonio Rimachi Hidalgo, François Isnaldo Dias Caldeira, Silvana Regina Perez Orrico, Fabio Renato Manzolli Leite, Raquel Mantuaneli Scarel-Caminaga

**Affiliations:** 1 Department of Diagnosis and Surgery, School of Dentistry, São Paulo State University—UNESP, Araraquara 14801-385, SP, Brazil; marco.rimachi@unesp.br (M.A.R.H.); francois.isnaldo@unesp.br (F.I.D.C.); silvana.orrico@unesp.br (S.R.P.O.); 2 Department of Morphology, Genetics, Orthodontics and Pediatric Dentistry, School of Dentistry, São Paulo State University—UNESP, Araraquara 14801-385, SP, Brazil; 3 Advanced Research Center in Medicine, Union of the Colleges of the Great Lakes (UNILAGO), São José do Rio Preto 15030-070, SP, Brazil; 4 National Dental Research Institute Singapore, National Dental Centre Singapore, Singapore 168938, Singapore; fabio@duke-nus.edu.sg; 5 Oral Health Academic Clinical Programme, Duke-NUS Medical School, Singapore 169857, Singapore

**Keywords:** periodontitis, PPAR-gamma, translational study, review, meta-analysis

## Abstract

**Aim:** Peroxisome Proliferator-Activated Receptor Gamma (PPAR-γ) plays important anti-inflammatory roles, including in periodontitis. This systematic review with a meta-analysis compiles evidence on the transcriptional and translational levels of PPAR-γ in clinical and experimental periodontitis studies, alongside functional enrichment and PPAR-γ interaction network analyses. **Method:** Electronic searches were conducted in six databases for publications up to February 2024. For the meta-analysis of experimental studies of periodontitis, PPAR-γ levels in the periodontal tissues were assessed through gene expression (RT-qPCR) or protein expression (Western blotting). In the clinical periodontitis studies, PPAR-γ levels in the gingival tissues were evaluated through protein expression (immunohistochemistry). A risk of bias (RoB) assessment was performed using the Systematic Review Centre for Laboratory Animal Experimentation (SYRCLE) and Newcastle Ottawa Scale (NOS) tools for experimental and clinical studies, respectively. The enrichment analysis was performed using the g:Profiler tool, and gene interaction networks were analyzed using GeneMANIA. **Results:** The meta-analysis demonstrated significantly lower PPAR-γ protein levels in the periodontal tissues from animals with periodontitis. *PPARG* mRNA and PPAR-γ quantification through immunohistochemistry remained inconclusive. The bioinformatics analyses indicated direct or indirect PPAR-γ-associated molecules involved in the immune response to periodontitis. The PPAR-γ protein expression was higher in periodontal tissues from healthy animals compared to that from those with periodontitis. **Conclusions:** Given the inconclusive findings of RT-qPCR and immunohistochemistry, further *PPARG* mRNA and PPAR-γ protein evaluations are needed to clarify their levels in the periodontal tissues.

## 1. Introduction

Periodontitis is a chronic inflammatory disease that develops due to an excessive inflammatory response in the host to the local microbiome [1]. This process is poorly controlled in susceptible individuals, leading to destruction of the periodontium [2]. The periodontal tissue damage generated is attributed mainly to the immune host’s response and not to direct microbial action and its products [3]. The observed bone loss is mediated largely by T helper 17 (TH17) cell-derived IL-17, which induces the expression of Receptor Activator of NF-κB ligand (RANKL) in the osteoblasts, leading to osteoclast activation and bone resorption [4]. In recent decades, significant studies have greatly advanced and improved our understanding of the pathogenesis and pathophysiology of this periodontitis, with the main achievement being the integration of different periodontal conditions into a single classification system [3]. This robust system considers important features such as the rate of progression and disease staging, allowing for the development of personalized therapeutic approaches [3,5]. Moreover, numerous epidemiological investigations have highlighted the influence of access to dental care, socioeconomic status, tobacco use, environmental exposure, and genetic predisposition [3,5].

A growing body of evidence suggests an association between periodontitis and various diseases, such as dyslipidemia [5], diabetes mellitus [6], hypertension [7], cardiovascular diseases [8], Alzheimer’s disease [9], and obesity [10]. Experimental and clinical studies have aimed to investigate the key markers associated with the health and/or disease statuses associating the co-occurrence of these conditions.

Peroxisome proliferator-activated receptors (PPARs) are transcription factors belonging to a broad family of ligand-activated nuclear hormone receptors within the steroid receptor superfamily [11]. PPARs are ligand-activated transcription factors that regulate genes essential for cellular differentiation and a range of metabolic processes [12]. The PPAR protein family is composed of three distinct subtypes, PPAR-α (NR1C1), PPAR-β/δ (NR1C2), and PPAR-γ (NR1C3), each originating from a different gene. The subtypes differ in terms of the ligands that activate them and the tissues in which they are predominantly expressed [11]. Despite having a very similar structure and binding to the same DNA sequence, these three isoforms perform unique physiological functions. Among them, PPAR-gamma (PPAR-γ) is particularly interesting due to its regulation of glucose metabolism, adipogenic differentiation [13], and the inflammatory response [14]. PPAR-γ activation occurs when a specific ligand binds to its receptor. This binding induces a conformational change in PPAR-γ which allows it to dissociate from co-repressor proteins. Subsequently, the activated PPAR-γ joins with another nuclear receptor, the RXR (retinoid X receptor), to form an active heterodimer complex. This PPAR-γ/RXR complex can bind to specific DNA sequences, known as PPREs (peroxisome proliferator response elements), to regulate the transcription of target genes such as nuclear factor of kappa light polypeptide gene enhancer in B-cell inhibitor, alpha (NFKBIA) and glucose transporter type 4 (GLUT-4) [11,12,13,14,15]. Given its important role in interacting with DNA at specific sites, PPAR-γ has aroused the interest of researchers based on its involvement in the management of metabolism and inflammation.

Clinical studies have demonstrated that dysregulation of PPAR-γ is related to the development of type 2 diabetes mellitus (T2DM), atherosclerosis [16], osteoporosis [17], and Alzheimer’s disease [18]. Experimental periodontitis studies have demonstrated that PPAR-γ plays an anti-inflammatory role by attenuating the production of pro-inflammatory cytokines, mitigating periodontal tissue destruction [19]. PPAR-γ is encoded by the *PPARG* gene, and some polymorphisms in this gene have been associated with periodontitis [20] and T2DM as a comorbidity of periodontitis in clinical studies [21].

It is essential to clarify the role of PPAR-γ in the pathogenesis of periodontitis, but this is complicated by limited and contradictory studies evaluating the gene and protein expression levels of PPAR-γ in the context of this disease. To address this, as made by others [22,23,24], we included clinical and experimental periodontitis studies in a systematic review with a meta-analysis, with the original aim of evaluating the expression at transcriptional levels of the *PPARG* gene and translational PPAR-γ protein levels. In addition, a bioinformatics approach was used to identify genes linked to PPAR-γ and their functions in the pathogenesis of periodontitis.

## 2. Material and Methods

### 2.1. Protocol Registration, the Focused Question, and PECO

The present systematic review (qualitative analysis) was conducted in accordance with the Preferred Reporting Items for Systematic Reviews and Meta-Analyses (PRISMA) statement [25], and the protocol was registered in the International Prospective Register of Systematic Reviews (PROSPERO—CDRD 42023470704). The focused question was “What are the gene or protein expression levels of Peroxisome Proliferator-Activated Receptor Gamma (PPAR-γ) among people or animals affected by periodontal diseases compared to those in a healthy state?”. PECO was as follows:

Population: People and/or animals (with periodontitis).

Exposure: Periodontal disease.

Comparison: People or animals in a healthy state.

Outcome: mRNA levels of the *PPARG* gene and/or PPAR-γ protein expression. Functional enrichment and interaction network of the identified genes and/or proteins.

### 2.2. The Eligibility Criteria

Inclusion criteria: The types of original studies selected for this review were clinical (human) and experimental (murine) periodontitis studies published as the full text or as an abstract only and without restrictions on the year of publication or language. We selected studies enrolling persons with periodontitis, regardless of ethnicity, sex, or age. The number of participants, sex, mean age, and periodontal clinical criteria for their selection were documented accordingly. Animals (rats or mice, without restrictions on strain) submitted to experimental periodontitis, regardless of sex or age, were also included in this study. The sample size, sex, mean age, and strategies for the induction of periodontitis were documented accordingly.

Considering the types of exposure, we selected studies that enrolled people affected only by periodontitis (any stages/grades of periodontitis) and without any systemic disease [26]. Similarly, we selected studies in which animals were submitted only to experimental periodontitis using ligature or inoculation with specific Gram-negative bacteria or lipopolysaccharide (LPS), without any other interventions. Studies were included where there were results on the PPAR-γ mRNA and/or protein levels and that were consistent with the methodology applied and the appropriate statistics for the variables.

Exclusion criteria: We excluded studies involving individuals or animals with systemic diseases or interventions that did not have a periodontitis group (without any associated comorbidity) and a control group. Also, studies without PPAR-γ mRNA or protein data were excluded. Review articles, letters to the editor, short communications, and publications in proceedings were also omitted.

### 2.3. The Strategy for the Identification of Studies

Searches were conducted in the following databases, from their oldest records to 18 February 2024: Medline through PubMed, Embase through Elsevier, Web of Science, Scopus, BVS (Virtual Health Library), and CINAHL through EBSCO. To explore the gray literature, the first 15 tabs on Google Scholar were used, and manual searches were performed in the reference lists of the included articles to identify potential studies reporting the gene and/or protein expression of PPAR-γ in periodontitis.

The search strategy involved the use of MESH, EMTREE, and DEC terms suitable for each specific database, combining keywords, text words, and search strings derived from the review question. These elements were combined using Boolean operators (AND/OR) to create a comprehensive search query aiming to retrieve relevant records for the review. Based on the PECO strategy, the descriptors used were “periodontitis”, “periodontal diseases”, “periodontal disease”, “chronic periodontitis”, “periodontal pocket”, “tooth loss”, “periodontal attachment loss”, “periodontal atrophy”, “alveolar bone loss”, “disease, periodontal”, “diseases, periodontal”, “clinical attachment loss”, “peroxisome proliferator-activated receptors”, “PPAR gamma”, “proliferator-activated receptors, peroxisome”, “receptors, peroxisome proliferator-activated”, PPAR, PPARG, PPAR-γ, NR1C3, PPARG1, PPARG2, and PPARgamma. Then, the strategy was tailored to meet the requirements of each database (Supplementary Table S1).

Manual searches were performed on the subject in various journals identified through the articles included in the systematic review, such as the Journal of Periodontal Research, Journal of Applied Oral Science, PPAR Research, Nigerian Journal of Clinical Practice, Journal of Hard Tissue Biology, International Immunopharmacology, and Journal of Ethnopharmacology.

### 2.4. The Article Screening and Data Extraction Processes

The bibliographic search results from all databases were exported into EndNote™ X8 (Thomson Reuters, New York, NY, USA) to eliminate duplicate references. Two researchers independently carried out the initial search to evaluate the titles and abstracts, with their results cross-checked for agreement. The full text of articles considered relevant based on the title and abstract was read independently and evaluated against the inclusion criteria. In cases of disagreements, a third researcher made the final decision. Rayyan software (https://www.rayyan.ai) [27] was used to manage article selection conflicts. Finally, two researchers independently reviewed all studies and extracted the data into a standardized table in Microsoft Excel.

The following information was extracted from each study included in this review:

(1) Clinical studies: (i) Authors, year of publication; (ii) the number of individuals in each group; (iii) age and sex; (iv) the biological material evaluated (gingival tissue, saliva, or blood); (v) biological molecular method of evaluation (RT-qPCR, ELISA, multiplex, Western blotting); and (vi) the gene or protein expression values in the unit of measurement originally published.
(2) Experimental periodontitis studies: (i) Authors, year of publication; (ii) the number of animals in each group; (iii) age, weight, sex; (iv) strain; (v) method of periodontitis induction (ligature, LPS, or bacteria); (vi) biological material evaluated (gingival tissue, periodontal ligament, blood); (vii) biological molecular method of evaluation (RT-qPCR, ELISA, multiplex, Western blotting); and (viii) values of gene or protein expression in the unit of measurement originally published.
(3) Mean and standard deviation data for the PPARG gene and PPAR-γ protein expression levels provided by the primary studies were utilized in the meta-analyses. When mean and standard deviation information was absent, graphs containing the PPAR-γ expression information were copied into a PowerPoint file. A ruler with a scale of increments of 0.05, created using the GraphPad Prism software (version 8.0.1), was aligned with the proportions of the copied figure scales. Horizontal lines were added to measure the mean and standard deviation sizes for each graph.

### 2.5. The Meta-Analysis

Only studies using the same assay method were included in each meta-analysis. For experimental periodontitis studies, the PPAR-γ levels in the periodontal tissues were assessed through the gene expression (RT-qPCR) or protein expression (Western blotting). For clinical periodontitis studies, PPAR-γ levels in the gingival tissues were evaluated through the protein expression (immunohistochemistry). The effect size was estimated and reported as the mean difference (MD), with a 95% confidence interval (CI) calculated for each mediator. The pooled effect was considered significant if the two-sided *p*-value was <0.05. An *I*^2^ *>* 50% indicated heterogeneity, and then the random effects model (the DerSimonian–Laird method) was applied; otherwise, the fixed model (the Mantel–Haenszel method) was used [28]. RevMan 5.4.1 (The Nordic Cochrane Centre, The Cochrane Collaboration, Copenhagen, Denmark, 2011) was used to pool the data and produce forest plots.

### 2.6. The Bioinformatics Analysis of Functional Enrichment and Interaction Networks of PPAR-γ

To assess the functional enrichment of the identified genes, the g:Profiler (https://biit.cs.ut.ee/gprofiler/gost accessed on 17 July 2025) public program was used, which accessed omics databases to establish protein–protein interactions. The significant threshold was set using the Benjamini–Hochberg false discovery rate (FDR) [29], with a *p*-value cutoff of 0.05. The analyzed data was verified on 4 April 2024.

To predict the potential association of periodontitis with PPAR-γ and other molecules, a gene interaction network was constructed using the Cytoscape program (version 3.10.1) with the GeneMANIA platform extension. GeneMANIA builds functional gene predictions through a Multiple Association Network Integration Algorithm, utilizing various publicly available datasets, such as genetic and protein–protein interaction networks [30,31]. The genes included in this review had to be evaluated through analytical methods such as Western blot, RT-qPCR, a microarray, immunohistochemistry, and multiplex assays. 

The functional analysis was conducted using a tool which maps genes to known functional information sources and detects significantly enriched terms, as well as analyzing genes resulting from high-throughput genomic data mining. This approach aggregates information from Gene Ontology, biological pathways, regulatory motifs in DNA, protein databases, and human phenotype ontology.

The generated network analysis organizes the information on the provided genes and the new genes identified based on their biological connections, creating a network of co-expression, physical interactions, co-localization, shared protein domains, predicted communication, and genetic interactions. Additionally, molecules marked in black represent the significant ones found in the articles included in this systematic review on PPAR-γ. These insights become valuable when used to prioritize future markers and their biological relationships in the context of periodontitis.

### 2.7. Quality Assessment

Any disagreement was discussed with a third researcher (R.M.S.C.). The methodological quality of the experimental periodontitis studies was evaluated using the Systematic Review Centre for Laboratory Animal Experimentation (SYRECLE’s) risk of bias (RoB) tools. The SYRECLE tool assesses bias such as selection, performance, detection, attrition, and reporting bias and other sources of bias [32]. The RoB for each domain was categorized as high if one or more criteria were not met, unclear if one or more criteria were partly met, and low if all criteria were met. Regarding the clinical periodontitis studies, the Newcastle Ottawa Scale (NOS) was used to evaluate the study quality. The NOS assesses three aspects: the selection of the study groups, the comparability of the study groups, and the assessment of exposure or outcomes. Studies with scores of 0–3 indicated low quality, 4–6 moderate quality, and 7–9 high quality.

## 3. Results

### 3.1. Results of the Study Selection

**Figure 1:**
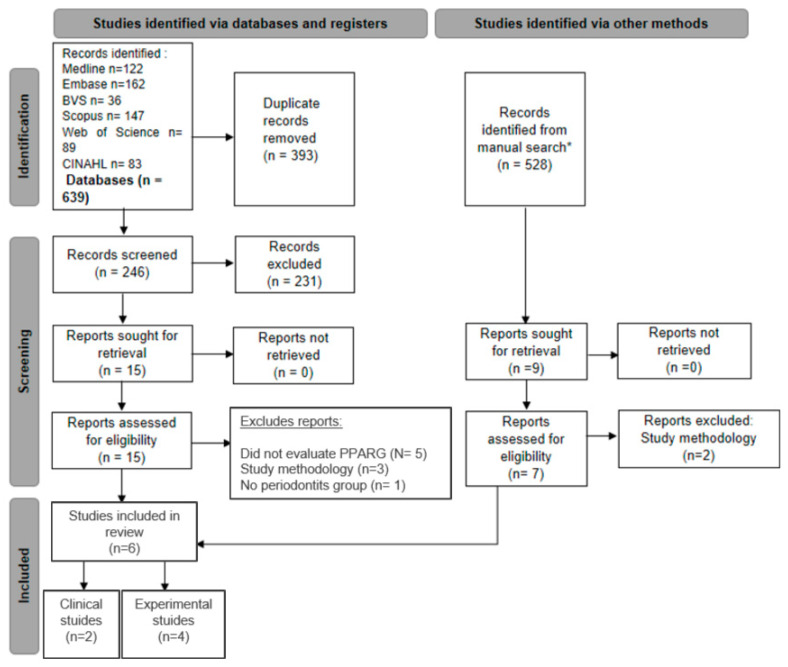
Studies included in the qualitative assessment based upon the guidelines of the PRISMA (Preferred Reporting Items for Systematic Review and Meta-analyses) flowchart (* gray literature search on Google Scholar).

The search strategy identified 639 studies. After removing duplicates, 246 unique studies remained. After the evaluation of their titles and abstracts, 231 were excluded since they were in vitro studies or reviews or did not evaluate PPAR-γ. Therefore, 15 articles were selected for full-text reading, after which only 6 were included in the current review. The reasons for excluding these articles are detailed in Figure 1.

The search for gray literature on Google Scholar identified 528 articles. After reading the titles and abstracts, nine articles were selected for full-text reading. Of these, six were already included in the initial database search, and two were excluded (Figure 1).

The selected studies were published between 2017 and 2024 and included four experimental periodontitis studies (animal models) [33,34,35,36] and two clinical periodontitis studies [37,38]. Amongst the four experimental studies, three used rats [34,35,36], and one used mice [33] (Table 1). The clinical studies involved systemically healthy, non-smoking individuals, with similar distributions of men and women in both the control and periodontitis groups [37,38]. The number of animals used in the experiments ranged from 6 to 8 for the control and 8 to 12 for the intervention groups [33,34,35,36]. The clinical studies included 14–15 participants in both the healthy and periodontitis groups [37,38]. More details are presented in Table 1.

### 3.2. The Induction of Experimental Periodontitis in the Animal Models and the Clinical Parameters for Selecting Patients

In one study, experimental periodontitis was induced by placing a 4-0 silk suture thread around the first molars [34]. Another study soaked a 7-0 silk suture thread in *Porphyromonas gingivalis* (*P.g.* strain ATCC 33277) solution [33]. A combination of an injection of 30 µL of lipopolysaccharide (LPS) solution into the gingival sulcus and ligature wires (0.2 mm in diameter) was used in one study [35]. Finally, an orthodontic thread (0.2 mm in diameter) soaked in a *P.g.* (strain W83) solution for 1 hour was used around the upper first molars [36] (Table 1).

In the clinical studies, the criteria used to select individuals with periodontitis were stage 3 and grade B [1,26]. In addition, clinical parameters such as the plaque index (PI), the gingival index (GI), and periodontal pocket depth (PPD) were assessed [37,38] (Table 1).

### 3.3. Study Characteristics

#### 3.3.1. Translational Assessment of *PPARG* and PPAR-γ in Experimental and Clinical Periodontitis Studies

Table 1 summarizes the original *PPARG* mRNA and PPAR-γ protein expression results reported in each study. Table 1 also shows the translational assessment of *PPARG* and PPAR-γ in the experimental (animal models) and clinical (human) periodontitis studies. With the exception of the *PPARG* mRNA levels found by Chen et al. using RT-qPCR [33], all of the other data were statistically different between the periodontitis and control groups, as reported by the original articles.

In the qualitative analysis, higher levels of *PPARG* mRNA were observed in healthy gingival tissues (without periodontitis) in both humans [37] and animals [33,36] with the protein levels measured through Western blotting, showing concordant and statistically significant higher PPAR-γ protein levels in healthy periodontal tissues. Regarding the protein expression of PPAR-γ in immunostained cells, in the experimental periodontitis study [34], a lower PPAR-γ protein expression was observed in the control group compared to that in the periodontitis groups. In the clinical studies involving gingival tissue biopsies, divergent results were reported. Taskan and Gevrek [38] demonstrated a higher number of PPAR-γ-immunostained cells in gingival tissues from individuals with periodontitis, while Karatas et al. [37] found the opposite.

#### 3.3.2. A Meta-Analysis of the PPAR-γ Expression Levels

**Figure 2:**
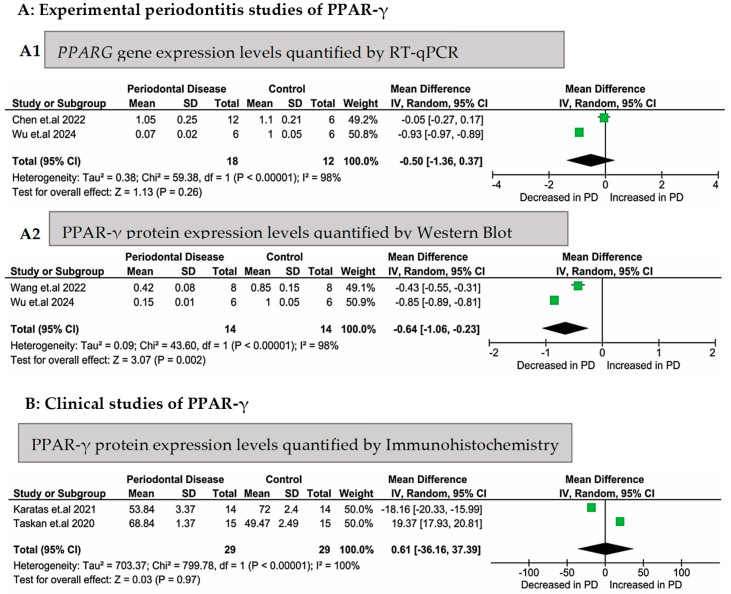
Meta-analyses of PPARG gene expression or PPAR-γ protein expression levels in experimental (animal) and clinical (human) periodontitis studies. Legend: (**A1**) [33,36] experimental periodontitis studies (in rodents) that evaluated gene (RT-qPCR) and protein [35,36] ((**A2**), Western blotting) expression levels in the periodontal tissues; [37,38] (**B**) clinical (human) studies that evaluated the protein expression levels (through immunohistochemistry) in gingival biopsies.

Figure 2 presents a meta-analysis evaluating the gene and protein expression levels of PPAR-γ in animals or humans with periodontitis compared to those in healthy controls. Importantly, data from human and animal studies were not combined at any stage, including during the data selection and the execution of the meta-analyses. In the experimental periodontitis studies, the gingival tissue from healthy animals exhibited higher mRNA levels (Z = 1.13), but without a statistically significant difference; *p* = 0.26, Figure 2(A1)). Regarding the PPAR-γ protein expression levels measured using Western blot in gingival tissues from rats, these levels were significantly higher in healthy animals (Z = 3.07; *p* = 0.002, Figure 2(A2)). Significant heterogeneity was found for both mRNA and protein levels (*p* < 0.00001; I^2^ = 98%).

The meta-analysis of the PPAR-γ protein expression levels evaluated through immunohistochemistry, which included two clinical studies, showed no statistically significant difference in the gingival tissues between the 29 subjects in the control group and the 29 subjects in the periodontitis group (Z = 0.03; *p* = 0.97, Figure 2B). Complete heterogeneity (I^2^ = 100%) was found for these two studies (*p* < 0.00001). 

#### 3.3.3. The Functional Enrichment Analysis

**Figure 3:**
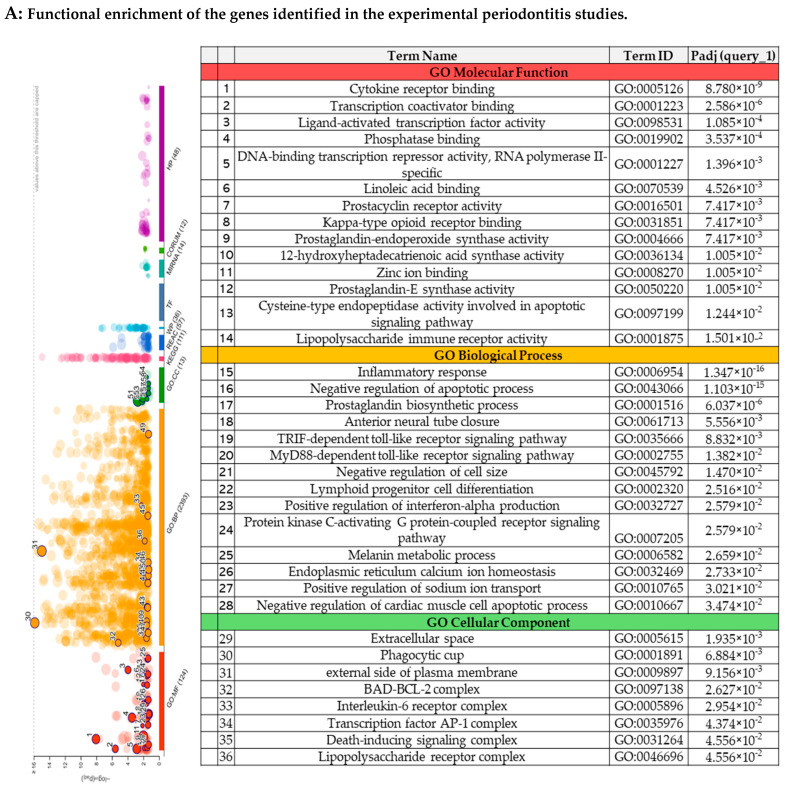

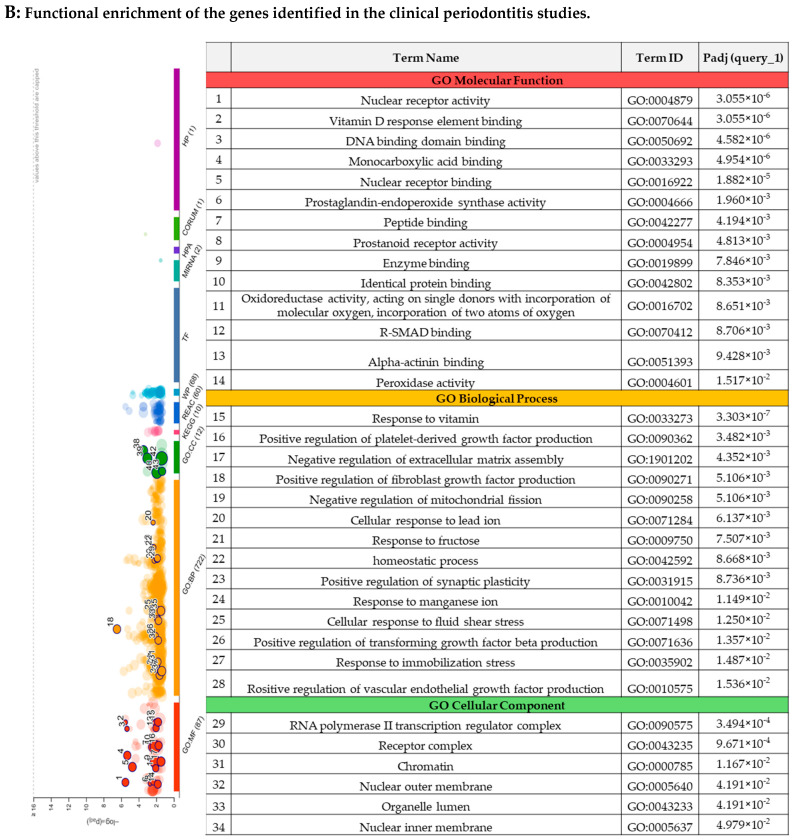
The functional enrichment of the genes identified in the systematic reviews.

Figure 3A demonstrates the functional enrichment of molecules evaluated in the experimental periodontitis studies. The Gene Ontology (GO) enrichment analyses demonstrated key molecular functions, such as cytokine receptor binding, transcription coactivator binding, and ligand-activated transcription factor activity. The biological processes involved included the immune–inflammatory response, the TRIF-dependent toll-like receptor signaling pathway, and positive regulation of interferon-beta production. Cellular components were significantly associated with the interleukin-6 receptor complex, the transcription factor AP-1 complex, and the lipopolysaccharide receptor complex.

The functional enrichment of the molecules evaluated in the clinical studies included here is represented in Figure 3B. The main molecular functions identified were nuclear receptor activity, the DNA binding domain, and prostaglandin-endoperoxide synthase activity. Analyses of the biological processes highlighted negative regulation of extracellular matrix assembly and monocyte differentiation. Regarding the cellular components, these genes were associated with the RNA polymerase II transcription regulator complex, the receptor complex, and chromatin.

#### 3.3.4. The Gene Network Analysis

**Figure 4:**
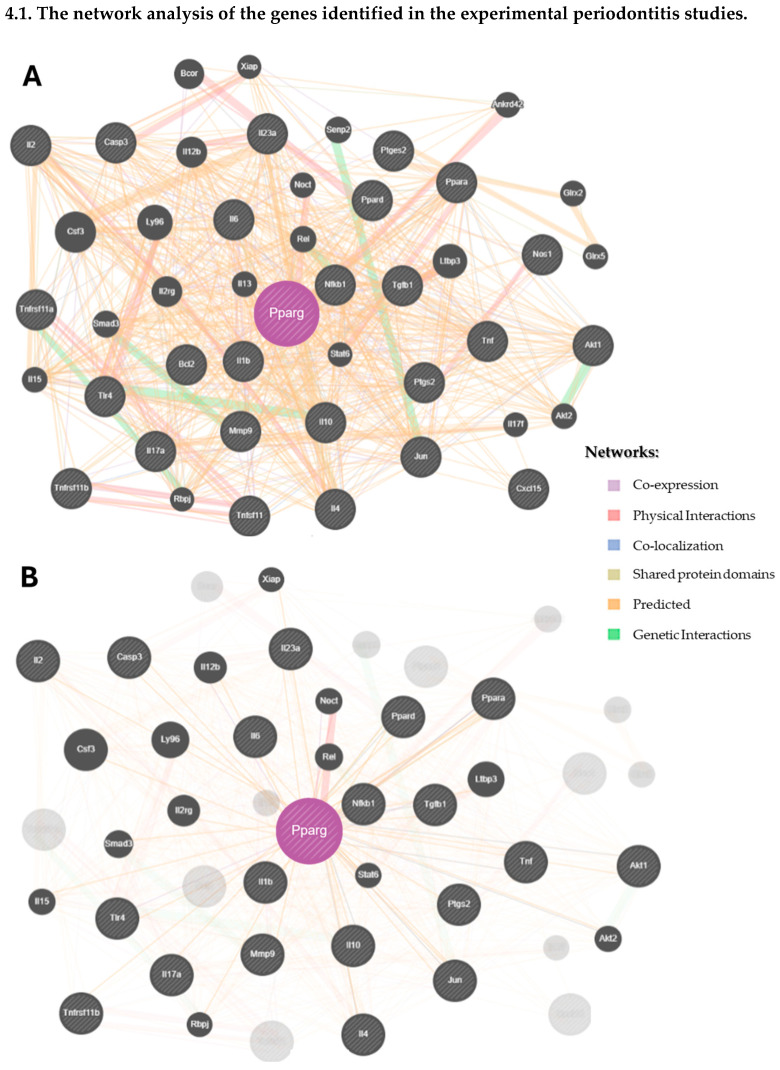

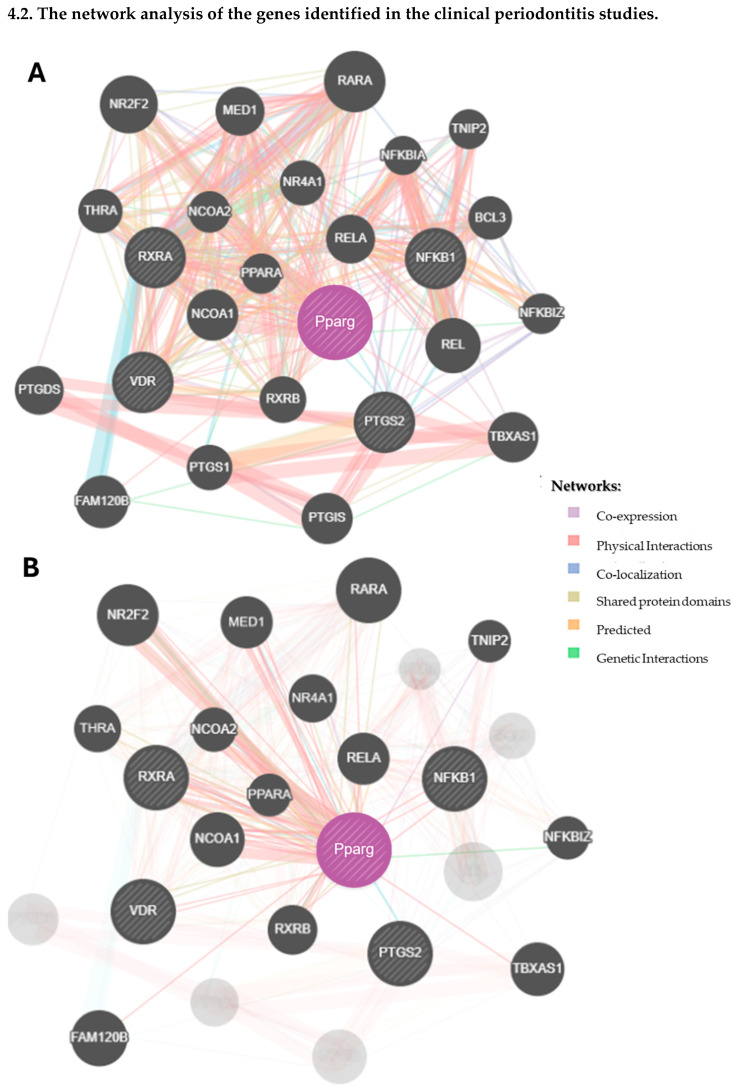
The network analysis of the genes identified in the systematic reviews.

The network analysis carried out using GeneMANIA is illustrated in Figure 4. For the experimental periodontitis studies, 20 new genes were identified, mainly through co-expression and physical interactions (Figure 4(1A)), and subsequently evaluated for their biological roles and their relationships with periodontitis (Supplementary Table S2). These molecules seem to actively participate in immune responses. Furthermore, from the types of gene–gene interactions, 39.56% were predicted, 26.88% were co-expression relationships, and 12.83% were physical interactions (Supplementary Table S3). Figure 4(1B) shows the physical interactions of PPAR-γ with molecules from the experimental periodontitis studies and with new molecules from the network analysis.

Similarly, Figure 4(2A) represents the network analysis from the clinical studies, where 20 new molecules were identified, many of which are involved in the immune system’s response to periodontitis (Supplementary Table S4). The main gene–gene interactions were physical (76.64%), 8.01% were co-expression, and 5.37% were predictive (Supplementary Table S5). Figure 4(2B) illustrates the specific physical interactions of PPAR-γ with other molecules from the network analysis.

#### 3.3.5. Quality Assessment of Studies

**Figure 5:**
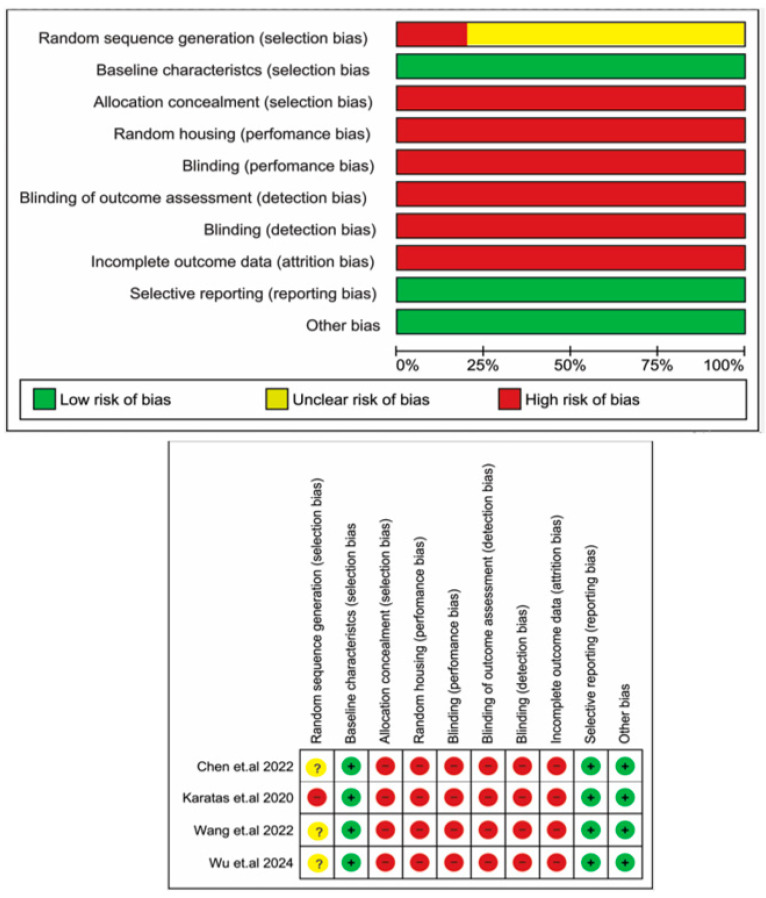
Quality assessment of the included studies according to the risk of bias tools for experimental periodontitis studies—SYRECLE’s RoB tool [33,34,35,36].

All of the clinical periodontitis studies were considered to be of high quality (a NOS score of 8) (Supplementary Table S6). The risk of bias assessment using the SYRECLE’s RoB tool, presented in Figure 5, showed that for “Random sequence generation”, only one study [34] had a high risk of bias, while three studies had an unclear risk of bias [33,35,36]. Other domains, including “Allocation concealment”, “Random housing”, “Blinding (performance bias)”, “Blinding of outcome assessment”, “Blinding (detection bias)”, and “Incomplete outcome data”, were classified as high-risk in all studies. Finally, domains such as “Baseline characteristics”, “Selective reporting”, and “Other bias” were rated as having a low risk of bias.

## 4. Discussion

In this systematic review, the literature on the mRNA and protein expression levels of PPAR-γ in patients and/or animals with periodontitis was investigated. Bioinformatics analyses were also conducted to understand the role of PPAR-γ in conjunction with other molecules in the pathogenesis of periodontitis better. The qualitative analysis (Table 1) of the experimental and clinical periodontitis studies demonstrated higher levels of *PPARG* mRNA in tissues from individuals and animals in the control groups. The quantitative analysis (Figure 2(A1)) demonstrated that even though the higher mRNA levels in the control groups in the two experimental studies were concordant, these findings did not reach statistical significance, maybe influenced by the high heterogeneity between them. Otherwise, a significantly increased PPAR-γ protein expression was observed in the control animals (Figure 2B). This can be explained by the absence of inflammation in these organisms.

In periodontitis, the abundant presence of bacterial LPS induces the activation of numerous inflammatory cells, as well as the production of nitric oxide [37]. In periodontitis-affected tissues, the beneficial effect of *PPARG* expression is regulation of the inflammatory response through the suppression of various inflammatory mediators, such as interleukin-1 beta (IL-1β), IL-6, IL-8, tumor necrosis factor-alpha (TNF-α), prostaglandin E2, and matrix metalloproteinases (MMPs). In vitro studies with human gingival fibroblasts have demonstrated that *PPARG* expression can reduce nuclear factor kappa B (NF-κB) expression by inhibiting IκB kinase activity, preventing NF-κB’s translocation to the nucleus, or by competing for limited amounts of transcriptional coactivators [39,40,41]. Therefore, PPAR-γ plays a crucial anti-inflammatory role, resulting in decreased tissue damage and inflammation caused by periodontal disease [19].

Despite the biological relevance of PPAR-γ in the context of periodontitis, the necessity of developing more studies in this area is evidenced by the scarce number of studies eligible for inclusion in the current review. Only one study was found that assessed the *PPARG* gene expression in humans [37], and two studies were found in animal models [33,36]. Given that PPAR-γ is a transcription factor, Western blotting and immunohistochemistry are the best methods for assessment. Regarding the PPAR-γ protein expression in the periodontal tissues, only two Western blotting studies in animal models could be included in the meta-analysis [35,36] (no Western blotting study was found in humans). As for immunohistochemistry, only Karatas et al. [34] employed this method in an animal model, while two studies made this kind of evaluation in humans [37,38]. Therefore, we identified a gap in the literature, highlighting the need for future clinical and experimental studies of periodontitis to understand the impact of the PPAR-γ transcription factor and its role in periodontitis better, either alone or in conjunction with other diseases, such as T2DM [20,21,42].

Sometimes, the relationship between the abundance of mRNA and proteins is complex and non-linear, varying significantly among proteins [43,44]. This variation can be explained by post-transcriptional regulation [45]. Interestingly, the meta-analyses presented concordance between the *PPARG* mRNA (Figure 2(A1)) and PPAR-γ protein levels (Figure 2(A2)) in the animal studies, even though the meta-analysis of *PPARG* mRNA did not reach statistical significance. Because these meta-analyses were composed of only two studies each, these findings could be different in future when the literature presents more studies evaluating both mRNA and protein levels to understand the role of PPAR-γ and related molecules and these results are interpreted without assuming complete concordance between them upfront [28].

Inconclusive findings were observed in this review as regarded the *PPARG* mRNA levels in the animal studies and the PPAR-γ protein levels assessed through immunohistochemistry in humans (Figure 2B). In rats submitted to periodontitis induction [34], a lower number of PPAR-γ-immunostained cells was observed in the alveolar bone and periodontal ligaments of the control animals compared to that in those submitted to ligatures. In the clinical studies, analyzing gingival tissue biopsies from subjects with stage 3 grade B periodontitis and periodontally healthy individuals (Table 1), Taskan and Gevrek [38] reported a significantly lower number of PPAR-γ-immunostained cells in the control group compared to that in the periodontitis group. However, the following study by the research group [37] presented the opposite results, despite them using the same anti-PPAR-gamma antibody (Abcam plc, Cambridge, UK), dilution (1:250), and H-score for immunohistochemistry. Both studies therefore employed the same methodology but yielded divergent results, as demonstrated in Figure 2B. There was heterogeneity in the populations that composed these clinical studies, as well as between the use of rats and mice, which may have introduced variability that contributed to the absence of significant differences in the mRNA levels and protein expression according to the immunohistochemistry analysis.

Combining bioinformatics tools with a systematic literature review has become increasingly common in medical sciences [46,47,48,49], although it remains scarce in the dental literature. This study is among the first to include bioinformatics for an analysis of functional enrichment and interaction networks in a systematic review.

The functional enrichment analysis using the g:Profiler tool [29] revealed significant components associated with biological processes, cellular components, and molecular functions. Enriched pathways in Gene Ontology indicated involvement in receptor binding of various cytokines (8.780 × 10^−9^), inflammatory processes (1.347 × 10^−16^), and IL-6 receptor complex activation (2.954 × 10^−2^). Recent studies have suggested that periodontitis, due to its inflammatory nature, triggers a broad axis of innate immunity. This activation occurs through various pro-inflammatory cytokines, such as IL-6, TNF-α, IL-1β, and IL-8, mediated by polymorphonuclear cells [50]. Dysregulated secretion of these mediators, both in terms of quantity and type, results in an inadequate immune response, causing significant tissue loss in the periodontium [50,51]

A gene–gene interaction network tool [30] was used to identify novel molecules potentially linked to PPAR-γ, verifying the association between the molecules described in the included studies. The main findings showed predictive functions in 39.56% of the molecules interconnected with PPAR-γ (considering the experimental periodontitis studies) and 76.64% physical interactions among the PPAR-γ molecules in the clinical studies. Importantly, most of the molecules found in the interaction networks were directly or indirectly related to the immune system.

Some limitations were noted in this study—first, a lack of restriction or stratification based on key variables such as age, sex, or ethnicity in the human studies or strain of rats or mice, sex, or age in the animal studies. This limitation occurred because there were few eligible studies, but it increased the heterogeneity of the results. Secondly, numerical data (mainly for *PPARG* mRNA levels) were absent from the original reports. Efforts were made to obtain additional information from the authors; however, these were unsuccessful. Another limitation was the low number of studies focusing on the PPAR-γ gene or protein expression in the context of periodontitis, highlighting the lack of assessments of the PPAR-γ protein expression in humans through Western blotting. Although the use of virtual scales to measure the mean and standard deviation in the mRNA and PPAR-γ protein levels in some studies was the only possible method for conducting qualitative analyses, it allowed us to perform a more precise and reliable meta-analysis. The use of available data from omics databases also presented challenges, as the datasets were relatively small and based on pre-existing data from the general and dental medical literature. Despite these limitations, this study accurately contributes to a clearer picture of the mRNA and protein levels of the important PPAR-γ transcription factor in the context of periodontitis and could stimulate future studies in this area.

## 5. Conclusions

This meta-analysis demonstrated higher levels of PPAR-γ protein expression in periodontal tissues from healthy animals compared to these levels in those affected by periodontitis. The functional enrichment analysis revealed important PPAR-γ mechanisms such as nuclear signaling and inflammatory responses associated with the pathogenesis of periodontitis. Further experimental and clinical studies are needed to confirm the findings observed here and to clarify *PPARG* mRNA and PPAR-γ protein levels through immunohistochemical evaluations, which will contribute to a comprehensive understanding of the role of this important molecule in periodontitis.

## Figures and Tables

**Table 1:** Characteristics of the experimental or clinical periodontitis studies included in this systematic review and the reported *PPARG* gene and PPAR-γ protein levels.

Author/Year	Organism	Control	Disease	Periodontitis Induction	RT-qPCR		Western Blotting		Immunohistochemistry	
		Sex/Number/ Mean Age	Sex/Number/ Mean Age		Periodontitis (Mean ± SD)	Control (Mean ± SD)	Periodontitis (Mean ± SD)	Control (Mean ± SD)	Periodontitis (Mean ± SD)	Control (Mean ± SD)
Chen et al., 2022 [33]	Wild-type (WT) C57BL/6 mice	Male; *n* = 6; 8–10 weeks	Male; *n* = 12; 8–10 weeks	7-0 silk ligature soaked in *P. gingivalis* around both maxillary second molars for 15 days.	1.05 ± 0.25 ^a^	1.1 ± 0.21 ^a^	-	-	-	-
Wu et al., 2024 [36]	Sprague-Dawley rats	Male; *n* = 6; 8 weeks	Male; *n* = 6; 8 weeks	0.2 mm diameter orthodontic wire soaked in *P. gingivalis W83 bacterial* (1 × 10^8^ CFU) around both maxillary first molars. Additionally, *P.g* LPS (50 µL, 2 mg/mL) was injected into the gingival crevices around the teeth. Duration = 4 weeks.	0.07 ± 0.02 ^a^	1.0 ± 0.05 ^b^	0.15 ± 0.01 ^a^	1.0 ± 0.05 ^b^	-	-
Wang et al., 2022 [35]	Sprague-Dawley rats	Male; *n* = 8; 6–8 weeks	Male; *n* = 8; 6–8 weeks	0.2 mm diameter fixed orthodontic treatment and 30 µL LPS injection into both maxillary second molars.	-	-	0.42 ± 0.08 ^a^	0.85 ± 0.15 ^b^	-	-
Karatas et al., 2020 [34]	Wistar rats	Female; *n* = 8; NR	Female; *n* = 8; NR	A 4-0 silk suture ligature around both lower first molars. The suture was removed from half of the rats on the 15th and 30th day.	-	-	-	-	P-L = 59.90 ± 7.12 ^a^ P-R = 64.03 ± 3.93 ^a^	23.06 ± 5.65 ^b^
**Author/Year**	**Organism**	**Control**	**Disease**	**Criteria for Subject Selection**	**RT-qPCR**		**Western Blotting**		**Immunohistochemistry**	
		**Sex/Number/** **Mean Age**	**Sex/Number/** **Mean Age**		**Periodontitis** **(Mean ± SD)**	**Control** **(Mean ± SD)**	**Periodontitis** **(Mean ± SD)**	**Control** **(Mean ± SD)**	**Periodontitis** **(Mean ± SD)**	**Control** **(Mean ± SD)**
Taskan and Gevrek, 2020 [38]	Human	Men/*n* = 8; Women/*n* = 7/45.05 ± 2.50 years	Men/*n* = 6; Women/*n* = 9/46.47 ± 1.89 years	Control: Never smokers and systemically healthy participants.	-	-	-	-	68.84 ± 1.37 ^a^	49.47 ± 2.49 ^b^
				Disease: Periodontitis stage 3 grade B. PI, GI, and PPD recorded in six sites per tooth. The mean value of six measurements was recorded.						
Karatas et al., 2021 [37]	Human	Men/*n* = 7; Women/*n* = 7/41.05 ± 1.80 years	Men/*n* = 7; Women/*n* = 7/42.35 ± 1.92 years	Control: Never smokers and systemically healthy participants.	0.1 ^a^	1 ^b^	-	-	53.84 ± 3.37 ^a^	72.0 ± 2.40 ^b^
				Disease: Periodontitis stage 3 grade B. PI, GI, and PPD recorded in six sites per tooth. The mean value of six measurements was recorded.						

NR: not reported; PI: plaque index; GI: gingival index; PPD: probing pocket depth; RT-qPCR: quantitative real-time PCR; SD: standard deviation. P-L: ligatures not removed on day 15. P-R: ligatures removed on day 15. Different letters indicate a statistically significant difference according to the original article.

## Data Availability

The data that support the findings of this study are available from the corresponding author upon reasonable request.

## Supplementary Materials

The following supporting information can be downloaded at: https://www.mdpi.com/article/10.3390/biomedicines13082028/s1, Table S1. Literature searches; Table S2. List of genes identified in the systematic review of PPAR-γ in the context of periodontitis in experimental periodontitis studies; Table S3. Categories of gene-gene interactions based on gene network analysis from experimental periodontitis studies; Table S4. List of genes identified in the systematic review of PPAR- γ in the context of periodontitis in Clinical studies; Table S5. Categories of gene-gene interactions based on gene network analysis of Clinical studies; Table S6. Quality assessment of the Clinical studies included in this systematic review using the Newcastle Ottawa scale.
