# Supplementary material for: A Systematic Review of Clinical and Experimental Periodontitis Studies Demonstrating the Expression of PPAR-Gamma: A Meta-Analysis and Bioinformatics Approach
Source: Biomedicines. 2025 Aug 20;13(8):2028. doi: 10.3390/biomedicines13082028 (PMC12383550; doi:10.3390/biomedicines13082028)
Supplement: Supplementary file 1 [file biomedicines-13-02028-s001.zip › biomedicines-3796917-supplementary.pdf]

Supplementary material

Supplementary Table S1. Literature searches

Database	**Literature searches**
PubMed/Medline	("periodontal"[All Fields] OR "periodontally"[All Fields] OR "periodontically"[All Fields] OR "periodontics"[MeSH Terms] OR "periodontics"[All Fields] OR "periodontic"[All Fields] OR "periodontitis"[MeSH Terms] OR "periodontitis"[All Fields] OR "periodontitides"[All Fields] OR "Periodontal Diseases"[All Fields] OR "Chronic Periodontitis"[All Fields] OR "Periodontal Pocket"[All Fields] OR "Tooth Loss"[All Fields] OR "Periodontal Attachment Loss"[All Fields] OR "Periodontal Atrophy"[All Fields] OR "Alveolar Bone Loss"[All Fields] OR "disease periodontal"[All Fields] OR "diseases periodontal"[All Fields] OR "Periodontal Disease"[All Fields] OR "Clinical Attachment Loss"[All Fields]) AND ("peroxisome proliferator activated receptors"[All Fields] OR "PPAR gamma"[All Fields] OR "peroxisome proliferator activated receptors"[All Fields] OR "proliferator activated receptors peroxisome"[All Fields] OR "receptors peroxisome proliferator activated"[All Fields] OR ("peroxisome proliferator activated receptors"[MeSH Terms] OR ("peroxisome"[All Fields] AND "proliferator activated"[All Fields] AND "receptors"[All Fields]) OR "peroxisome proliferator activated receptors"[All Fields] OR "ppar"[All Fields]) OR "peroxisome proliferator activated receptor"[All Fields] OR "peroxisome proliferator activated receptor"[All Fields] OR "proliferator activated receptor peroxisome"[All Fields] OR "receptor peroxisome proliferator activated"[All Fields] OR ("PPAR gamma"[MeSH Terms] OR ("ppar"[All Fields] AND "gamma"[All Fields]) OR "PPAR gamma"[All Fields] OR "ppargamma"[All Fields] OR "ppargamma s"[All Fields]) OR "peroxisome proliferator activated receptor gamma"[All Fields] OR "peroxisome proliferator activated receptor gamma"[All Fields] OR "PPARG"[All Fields] OR ("PPAR gamma"[MeSH Terms] OR ("ppar"[All Fields] AND "gamma"[All Fields]) OR "PPAR gamma"[All Fields]))
Embase	('periodontitis'/exp OR 'periodontal disease'/exp OR 'chronic periodontitis'/exp OR 'periodontal pocket'/exp OR 'alveolar bone loss'/exp OR 'tooth loss' OR 'periodontal attachment loss' OR 'periodontal atrophy' OR 'disease, periodontal' OR 'diseases, periodontal' OR 'clinical attachment loss') AND ('peroxisome proliferator activated receptor'/exp OR 'peroxisome proliferator activated receptor gamma'/exp OR 'peroxisome proliferator activated receptors' OR 'peroxisome proliferator-activated receptors' OR 'ppar' OR 'peroxisome proliferator activated receptor' OR 'ppar gamma' OR 'peroxisome proliferator activated receptor gamma' OR 'ppar-γ' OR 'pparg')

BVS	(periodontite OR periodontitis OR periodontitis OR "Doenças Periodontais" OR "enfermedades periodontales" OR "Maladies parodontales" OR "Periodontite Crônica" OR "Chronic Periodontitis" OR "Periodontitis Crónica" OR "Bolsa Periodontal" OR "Periodontal Pocket" OR "Bolsa Periodontal" OR "Perda de Dente" OR "Tooth Loss" OR "Pérdida de Diente" OR "Perda da Inserção Periodontal" OR "Periodontal Attachment Loss" OR "Pérdida de la Inserción Periodontal" OR "Atrofia Periodontal" OR "Periodontal Atrophy" OR "Atrofia Periodontal" OR "pérdida de hueso alveolar" OR "Perda do Osso Alveolar" OR "Résorption alvéolaire" OR "Perda Óssea Periodontal" OR "Reabsorção Alveolar" OR "Reabsorção Periodontal") AND ("Receptores Activados del Proliferador del Peroxisoma" OR "receptores activados por proliferadores de peroxisomas" OR "Peroxisome Proliferator-Activated Receptors" OR "Receptor Ativado por Proliferador de Peroxissoma" OR "Receptor Ativado por Proliferador de Peroxissomo" OR "Receptores Ativados por Proliferador de Peroxissoma" OR "NUC1 PPAR" OR "PPAR gamma" OR "Récepteur PPAR gamma" OR "PPAR gamma" OR "PPARgama" OR "PPAR-gama" OR "Receptor gama Ativado pelo Proliferador de Peroxissoma" OR "Receptor gama Ativado pelo Proliferador de Peroxissomas" OR "Receptor gama Ativado pelo Proliferador de Peroxissomos" OR "Receptor gama Ativado por Proliferador de Peroxissoma" OR "Receptor gama Ativado por Proliferador de Peroxissomas" OR "Receptor gama Ativado por Proliferador de Peroxissomo" OR "Receptor gama Ativado por Proliferador de Peroxissomos" OR "Receptor gama Ativado por Proliferadores de Peroxissomas" OR "Receptor gama Ativado por Proliferadores de Peroxissomos" OR "Receptores Ativados por Proliferadores de Peroxissoma gama" OR "Receptores gama Ativados por Proliferadores de Peroxissomas" OR "Receptores gama Ativados por Proliferadores de Peroxissomos") AND
Scopus	(TITLE-ABS-KEY (periodontitis OR "Periodontal Diseases" OR "Chronic Periodontitis" OR "Periodontal Pocket" OR "Tooth Loss" OR "Periodontal Attachment Loss" OR "Periodontal Atrophy" OR "Alveolar Bone Loss" OR "Disease, Periodontal" OR "Diseases, Periodontal" OR "Periodontal Disease" OR "Clinical Attachment Loss") AND TITLE-ABS-KEY ("Peroxisome Proliferator-Activated Receptors" OR "PPAR gamma" OR "Peroxisome Proliferator Activated Receptors" OR "Proliferator-Activated Receptors, Peroxisome" OR "Receptors, Peroxisome Proliferator-Activated" OR ppar OR "Peroxisome Proliferator-Activated Receptor" OR "Peroxisome Proliferator Activated Receptor" OR "Proliferator-Activated Receptor, Peroxisome" OR "Receptor, Peroxisome Proliferator-Activated" OR ppargamma OR "Peroxisome Proliferator-Activated Receptor gamma" OR " Peroxisome Proliferator Activated Receptor gamma" OR pparg OR ppar-γ))
Web Of Science	(TS=(Periodontitis OR "Periodontal Diseases" OR "Chronic Periodontitis" OR "Periodontal Pocket" OR "Tooth Loss" OR "Periodontal Attachment Loss" OR "Periodontal Atrophy" OR "Alveolar Bone Loss" OR "Disease, Periodontal" OR "Diseases, Periodontal" OR "Periodontal Disease" OR "Clinical Attachment Loss")) AND TS=("Peroxisome Proliferator-Activated Receptors" OR "PPAR gamma" OR "Peroxisome Proliferator Activated Receptors" OR "Proliferator-Activated Receptors, Peroxisome" OR "Receptors, Peroxisome Proliferator-Activated" OR PPAR OR "Peroxisome Proliferator-Activated Receptor" OR "Peroxisome Proliferator Activated Receptor" OR "Proliferator-Activated Receptor, Peroxisome" OR "Receptor, Peroxisome Proliferator-Activated" OR PPARgamma OR "Peroxisome Proliferator-Activated Receptor gamma" OR "Peroxisome Proliferator Activated Receptor gamma" OR PPARG OR PPAR-γ)

CINAHL	(Periodontitis OR "Periodontal Diseases" OR "Chronic Periodontitis" OR "Periodontal Pocket" OR "Tooth Loss" OR "Periodontal Attachment Loss" OR "Periodontal Atrophy" OR "Alveolar Bone Loss" OR "Disease, Periodontal" OR "Diseases, Periodontal" OR "Periodontal Disease" OR "Clinical Attachment Loss") AND ("Peroxisome Proliferator-Activated Receptors" OR "PPAR gamma" OR "Peroxisome Proliferator Activated Receptors" OR "Proliferator-Activated Receptors, Peroxisome" OR "Receptors, Peroxisome Proliferator-Activated" OR PPAR OR "Peroxisome Proliferator-Activated Receptor" OR "Peroxisome Proliferator Activated Receptor" OR "Proliferator-Activated Receptor, Peroxisome" OR "Receptor, Peroxisome Proliferator-Activated" OR PPARgamma OR "Peroxisome Proliferator-Activated Receptor gamma" OR" Peroxisome Proliferator Activated Receptor gamma" OR PPARG OR PPAR-γ)

Supplementary Table S2 List of genes identified in the systematic review of PPAR-γ in the context of periodontitis in experimental periodontitis studies.

Gene symbol	Gene name in NIH	Functions
**Biological function of PPAR-γ in Mus musculus**		
PPARG	Peroxisome proliferator activated receptor gamma	Acute inflammatory response<br>Cellular response to insulin stimulus<br>Cytokine-mediated signaling pathway
**Genes associated with PPAR-γ evaluated in animal model studies included**		
PPARA	Peroxisome proliferator activated receptor alpha	Ligand-activated transcription factor activity<br>Positive regulation of lipid metabolic process<br>Regulation of protein binding
PPARD	Peroxisome proliferator activated receptor delta	Collagen biosynthetic process<br>Vascular process in circulatory system<br>Insulin secretion
TNF	Tumor necrosis factor	Acute inflammatory response<br>Adaptive immune response<br>Cytokine-mediated signaling pathway
IL1B	Interleukin 1 beta	Cytokine production involved in immune response<br>NIK/NF-kappa B signaling<br>Acute inflammatory response
NFKB1	Nuclear Factor Kappa-B Subunit 1	Negative regulation of cytokine production<br>Negative regulation of defense response<br>Cellular response to biotic stimulus
IL10	Interleukin 10	Interleukin-6 production<br>Regulation of MHC class II biosynthetic process<br>B cell activation
IL6	Interleukin 6	Adaptive immune response<br>Cytokine production involved in inflammatory response<br>Alpha-beta T cell activation
IL2	Interleukin 2	B cell mediated immunity<br>Adaptive immune response<br>Alpha-beta T cell activation

IL23a	Interleukin 23 subunit alpha	Adaptive immune response<br>Alpha beta T cell activation<br>Positive regulation of adaptive immune response
CXCL8	Interleukin 8	Humoral immune response<br>Cytokine receptor binding<br>Antimicrobial humoral response
RANKL (TNFSF11)	Nuclear Factor Activator Receptor Kappa B (TNF Superfamily Member 11)	Bone resorption<br>I-kappa B kinase/NF-kappa B signaling<br>Osteoclast differentiation
OPG (TNFRSF11B)	Osteoprotegerin (TNF Receptor Superfamily Member 11b)	Regulation of bone remodeling<br>Bone resorption<br>Tissue remodeling
RANK (Tnfrsf11a)	Receptor Activator Of Nuclear Factor Kappa B (TNF Receptor Superfamily Member 11a)	Bone remodeling<br>Bone resorption<br>Cellular response to tumor necrosis factor,
IL17F	Interleukin 17F	Cytokine production involved in inflammatory response<br>Osteoclast differentiation<br>Production of molecular mediator of immune response
Ptgs2	Prostaglandin-Endoperoxide Synthase 2	Acute inflammatory response<br>Positive regulation of inflammatory response<br>Negative regulation of ion transport
PTGES2	Prostaglandin - E Synthase 2	
NOS1	Nitric Oxide Synthase 1	Insulin secretion<br>Negative regulation of cellular component movement<br>Negative regulation of protein transport
**Genes identified in gene network analysis**		
BCOR	BCL6 Corepressor	Biomineral tissue development<br>Biomineralization<br>Tooth mineralization
IL12B	Interleukin 12B	Activated T cell proliferation<br>Activation of protein kinase activity<br>Adaptive immune response
NOCT	Nocturnin	Fat cell differentiation<br>Osteoblast differentiation<br>Regulation of fat cell differentiation,
ANKRD42	Ankyrin Repeat Domain 42	Regulation of cytokine production involved in Inflammatory response<br>Cytokine production involved in inflammatory response<br>Positive regulation of defense response
CSF3	Colony Stimulating Factor 3	Cytokine receptor binding<br>Growth factor receptor binding<br>Regulation of protein binding

STAT6	Signal Transducer And Activator Of Transcription 6	Adaptive immune response<br>Alpha-beta T cell activation<br>B cell activation involved in immune response
IL2RG	Interleukin 2 Receptor Subunit Gamma	Positive regulation of B cell activation<br>Alpha-beta T cell activation<br>Alpha-beta T cell differentiation,
IL4	Interleukin 4	Activated T cell proliferation<br>Adaptive immune response<br>Alpha-beta T cell activation
IL15	Interleukin 15	Alpha-beta T cell activation<br>Cytokine-mediated signaling pathway<br>Receptor signaling pathway via JAK-STAT
GLRX5	Glutaredoxin 5	Iron homeostasis
IL17F	Interleukin 17F	Cytokine production involved in inflammatory response<br>Defense response to bacterium<br>Humoral immune response
BCL2	BCL2 Apoptosis Regulator	Apoptotic signal pathway in absence of ligand<br>Regulation of autophagy<br>Regulation of osteoblast proliferation
TLR4	Toll Like Receptor 4	B cell activation involved in immune response<br>Production of molecular mediator involved in inflammatory response<br>Regulation of IL1 production
IL17A	Interleukin 17A	Cytokine production involved in inflammatory response<br>IL1 beta production<br>Positive regulation of production of molecular mediator of immune response
MMP9	Matrix Metallopeptidase 9	Cellular response to oxidative stress<br>Regulation of fibroblast proliferation<br>Regulation of leucocyte migration
CASP3	Caspase 3	Activated T cell proliferation<br>Negative regulation of B cell activation<br>Tumor necrosis factor receptor superfamily binding
TGFB1	Transforming Growth Factor Beta 1	Adaptive immune response<br>B cell activation involved in immune response<br>Alpha-beta cell T cell differentiation involved in immune response
JUN	Jun Proto-Oncogene, AP-1 Transcription Factor Subunit	Leukocyte activation involved in inflammatory response<br>Macrophage activation<br>Cellular response to extracellular stimulus
AKT1	AKT Serine/Threonine Kinase 1	Cellular response to extracellular stimulus<br>Cellular response to oxidative stress<br>Fibroblast migration

LY96	Lymphocyte Antigen 96	Cellular response to molecule of bacterial origin<br>Positive regulation of TNF superfamily cytokine production<br>Toll-like receptor signaling pathway
LTBP3	Latent Transforming Growth Factor Beta Binding Protein 3	Bone remodeling<br>Bone mineralization<br>Regulation of bone resorption
SMAD3	SMAD Family Member 3	Regulation bone mineralization<br>Interleukin 1 beta production<br>Regulation of defense response
SENP2	SUMO Specific Peptidase 2	Regulation of protein binding<br>Regulation of mitotic cell cycle phase transition<br>Cysteine type endopeptidase activity
REL	REL Proto-Oncogene, NF-KB Subunit	Interleukin 12 production<br>Negative regulation of cytokine production<br>Regulation of IL12 production
GLRX2	Glutaredoxin 2	Response to reactive oxygen species
IL13	Interleukin 13	Cell activation involved in immune response<br>IL10 production<br>Leukocyte activation involved in immune response
AKT2	AKT Serine/Threonine Kinase 2	Cellular response to environmental stimulus<br>Negative regulation of endopeptidase activity<br>Positive regulation of intracellular transport
RBPJ	Recombination Signal Binding Protein For Immunoglobulin Kappa J Region	B cell activation<br>Dendritic cell differentiation<br>Endocrine system development
XIAP	X-Linked Inhibitor Of Apoptosis	Negative regulation of cysteine-type endopeptidase activity<br>Regulation of cysteine-type endopeptidase activity involved in apoptotic process<br>Regulation of Wnt signaling pathway

Supplementary Table S3 Categories of gene-gene interactions based on gene network analysis from experimental periodontitis studies.

Type of Interaction	Percentage of Overall Interaction
Predicted	39.56 %
Co-expression	26.88 %
Physical interactions	12.83 %
Other	12.47 %
Genetic Interactions	7.26 %
Co-localization	3.63 %
Shared protein domains	0.51 %

Supplementary Table S4 List of genes identified in the systematic review of PPAR- γ in the context of periodontitis in Clinical studies.

Gene symbol	Gene name in NIH	Functions
**Biological function of PPAR- γ in Clinical studies (Homo sapiens)**		
PPARG	Peroxisome proliferator activated receptor gamma	Regulation of inflammatory response<br>Regulation of lipid metabolic process<br>Intracellular receptor signaling pathway
**Genes associated with PPAR- γ evaluated in the Clinical studies included**		
RXRA	Retinoid X Receptor Alpha	Intracellular receptor signaling pathway<br>Transcription regulator complex<br>Ligand activated transcription factor activity
VDR	Vitamin D Receptor	Regulation of epithelial cells proliferation<br>Intracellular receptor signaling pathway<br>Regulation of keratinocyte differentiation
PTGS2	Prostaglandin-Endoperoxide Synthase 2	Positive regulation of lipid metabolic process<br>Regulation of inflammatory response<br>Blood vessel endothelial cell migration
NKFB	Nuclear Factor Kappa B	Cellular response to interleukin-1<br>Response to interleukin-6<br>Regulation of type I interferon production
**Genes identified in gene network analysis**		
RARA	Retinoic Acid Receptor Alpha	Intracellular receptor signaling pathway<br>Ligand activated transcription factor activity<br>Retinoid binding
NR2F2	Nuclear Receptor Subfamily 2 Group F Member 2	Ligand activated transcription factor activity<br>Regulation of epithelial cell proliferation<br>Retinoid binding
FAM120B	Family With Sequence Similarity 120 Member B	Intracellular receptor signaling pathway
PTGIS	Prostaglandin I2 Synthase	Negative regulation of defense response<br>Regulation of inflammatory response<br>Intracellular receptor signaling pathway
NCOA1	Nuclear Receptor Coactivator 1	Transcription co-activator activity<br>Response to extracellular stimulus<br>Cellular response to nutrient
TBXAS1	Thromboxane A Synthase 1	Arachidonic acid metabolic process<br>Fatty acid biosynthetic process<br>Prostaglandin biosynthetic process
MED1	Mediator Complex Subunit 1	Epithelial cell proliferation<br>Regulation of keratinocyte differentiation<br>Transcription co-activator activity
PTGDS	Prostaglandin D2 Synthase	Monocarboxylic acid biosynthetic process<br>Unsaturated fatty acid metabolic process<br>Intra-molecular oxidoreductase activity
RXRB	Retinoid X Receptor Beta	Intracellular receptor signaling pathway<br>Ligand-activated transcription factor activity<br>Transcription regulator complex

PTGS1	Prostaglandin-Endoperoxide Synthase 1	Arachidonic acid metabolic process<br>Unsaturated fatty acid metabolic process<br>Prostaglandin metabolic process
NR4A1	Nuclear Receptor Subfamily 4 Group A Member 1	Epithelial cell proliferation<br>Blood vessel endothelial cell migration<br>Nuclear receptor binding
THRA	Thyroid Hormone Receptor Alpha	Regulation of transcription initiation from RNA polymerase II promoter<br>Intracellular receptor signaling pathway<br>Ligand-activated transcription factor activity
NCOA2	Nuclear Receptor Coactivator 2	Nuclear hormone receptor binding<br>Organic hydroxy compound transport<br>Transcription co-activator activity
PPARA	Peroxisome Proliferator Activated Receptor Alpha	Negative regulation of defense response<br>Negative regulation of response to external stimulus<br>Positive regulation of fatty acid metabolic process
REL	REL Proto-Oncogene, NF-KB Subunit	Negative regulation of cytokine production<br>Regulation of type I interferon production<br>Type I interferon production
RELA	RELA Proto-Oncogene, NF-KB Subunit	Cellular response to interleukin-1<br>Regulation of type I interferon production<br>Positive regulation of defense response
BCL3	BCL3 Transcription Coactivator	Negative regulation of cytokine production<br>NIK/NF-kappa B signaling<br>Regulation of binding
BFKBIZ	NF-Kappa-B Inhibitor Zeta	Positive regulation of alpha-beta T cell differentiation<br>Positive regulation of CD4-positive<br>Positive regulation of defense response
TNIP2	TNFAIP3 Interacting Protein 2	Cellular response to interleukin-1<br>Response to interleukin-1<br>Response to lipopolysaccharide
NFKBIA	NFKB Inhibitor Alpha	Cellular response to interleukin-1<br>Regulation of inflammatory response<br>Positive regulation of defense response

Supplementary Table S5 Categories of gene-gene interactions based on gene network analysis of Clinical studies.

Type of Interaction	Percentage of Overall Interaction
Physical interactions	76.64 %
Co-expression	8.01 %
Predicted	5.37 %
Co-localization	3.63 %
Genetic Interactions	2.87 %
Pathway	1.88 %
Shared protein domains	0.60 %

Supplementary Table S6. Quality assessment of the Clinical studies included in this systematic review using the Newcastle Ottawa scale.

Author	Year	Criterion Scores			Total Score
		Selection	Comparability	Exposure	
Taskan	2020	***	**	***	8- high quality
Karatas	2021	***	**	***	8- high quality
